# Hsa_piR_016975 Is a Novel Target of Nanotherapy that Boosts Hepatoma Progression and Sorafenib Resistance by Abating Maspin/GPX4-Mediated Ferroptosis

**DOI:** 10.34133/bmr.0225

**Published:** 2025-07-02

**Authors:** Wei Feng, Jing Xu, Bairong Chen, Jibin Liu, Yuhao Hu, Xuemin Cao, Jing Qi, Linling Ju, Jianguo Shao, Peixin Dong, Lin Chen, Feng Wang

**Affiliations:** ^1^ Research Center of Clinical Medicine, Affiliated Hospital of Nantong University, Medical School of Nantong University, Nantong 226001, China.; ^2^ Institute of Oncology, Affiliated Tumor Hospital of Nantong University, Nantong 226631, Jiangsu, China.; ^3^ Nantong Institute of Liver Diseases, Nantong Third People’s Hospital, Affiliated Nantong Hospital 3 of Nantong University, Nantong 226006, China.; ^4^ Department of Obstetrics and Gynecology, Hokkaido University School of Medicine, Hokkaido University, Sapporo, Japan.

## Abstract

PIWI-interacting RNAs (piRNAs) are known to be involved in germline development, but their potential mechanisms in carcinogenesis remain elusive. Herein, we investigated the roles of hsa_piR_016975, a novel piRNA, in hepatocellular carcinoma (HCC) progression and its therapeutic effects on drug resistance to sorafenib. The results disclosed that hsa_piR_016975 was highly expressed in HCC and promoted HCC growth, metastasis, epithelial mesenchymal transition (EMT) formation, and sorafenib resistance. Mechanistic research uncovered that hsa_piR_016975 could target inhibition of the expression of serpin family B member 5 (SERPINB5; also known as Maspin) while up-regulating glutathione peroxidase 4 (GPX4) expression, thereby attenuating the ferroptosis and resulting in HCC progression and drug resistance. Furthermore, a novel delivery system was constructed, which was encapsulated with sorafenib and hsa_piR_016975 inhibitor in the nanoparticles of polylactic-co-glycolic acid and subsequently coated with the HCC cell membrane (namely, in-016975/Sora@PLGA-CM). The nanocomposites could effectively reverse HCC progression and sorafenib resistance by inducing hsa_piR_016975/Maspin/gpx4 axis-mediated ferroptosis in both subcutaneous xenograft model and orthotopic transplantation model. Overall, this study illuminates the critical role and molecular mechanisms of hsa_piR_016975 in hepatocarcinogenesis and provides a promising piRNA-oriented nanodelivery strategy for overcoming sorafenib resistance in HCC.

## Introduction

As one of the most common malignancies worldwide, hepatocellular carcinoma (HCC) owns the second highest mortality rate [1]. High trend of recurrence and metastasis markedly affects the prognosis of HCC patients [2,3]. With the expansion of first-line therapy options, sorafenib has been recommended for HCC patients by international guidelines over the past few decades. As an oral multitarget tyrosine kinase inhibitor (TKI), sorafenib inhibits tumor cell proliferation and promotes cell apoptosis mainly by inhibiting tyrosine kinase and downstream serine/threonine kinase activity [4]. However, according to statistics, only approximately 30% of HCC patients benefit from sorafenib treatment, and the vast majority of patients develop drug resistance within half a year [4,5]. Consequently, it is important to explore the underlying mechanisms of hepatocarcinogenesis and chemoresistance for improving the prognosis of HCC patients.

PIWI-interacting RNAs (piRNAs), a class of small noncoding RNAs, are approximately 24 to 35 nucleotides long. Its precursors originate from the transcription of genomic loci and are digested by endonucleases to form mature piRNAs [6,7]. Initially, many studies demonstrated that piRNAs are enriched in germline cells and participate in genomic silencing to maintain stable gene expression [8]. However, mounting documents have uncovered that dysregulated piRNAs widely participate in the onset and development of human malignancies. These piRNAs may conduce to cancer cell growth, metastasis, and drug resistance, revealing their critical roles in carcinogenesis [9]. Hence, it is vital to enunciate the specific mechanisms of these piRNAs involved in tumorigenesis and their diagnostic and therapeutic values for tumors.

Recent reports have shown that sorafenib can induce ferroptosis in HCC cells, revealing that sorafenib resistance may result from ferroptosis suppression in HCC [10,11]. As a new type of programmed cell death, ferroptosis is caused by glutathione peroxidase deficiency and reactive oxygen species (ROS) accumulation [12]. Moreover, glutathione peroxidase 4 (GPX4) has been confirmed to be a key regulatory factor in ferroptosis, and it is able to use glutathione to convert lipid peroxides (LPOs) into atoxic lipid alcohols, thereby reducing lipid deposition, unlocking its meaningful role in defending cells from oxidative stress [13]. Dike et al. [14] found that GPX4 could hasten prostate cancer progression while enhancing resistance to castration drugs. LncRNA PVT1 can regulate GPX4 expression through the miR-195-5p/PLAG1 axis, thereby inducing ferroptosis of HCC cells and enhancing the sensitivity to sorafenib [15]. However, whether piRNAs participate in HCC progression and sorafenib resistance via GPX4-mediated ferroptosis is still unclear.

With the development of genomic research, gene therapy has become a popular method for cancer treatment. RNA interference is a common method for regulating gene expression and facilitates the development of different molecular drugs. However, accurate and stable delivery of small interfering RNAs (siRNAs) into tumor cells is a challenge for researchers in gene therapy [16,17]. Recently, cell membrane-coated nanoparticles have been used to deliver siRNA into target cells and have shown great advantages compared to other delivery systems. This delivery system combines the properties of both cell membranes and nanoparticles and can accurately target the tumor site, release the drug consistently, prolong the half-life of blood circulation, and maximize the accumulation of therapeutic components at the tumor site [18–20]. Therefore, siRNA delivery via cell membrane-coated nanotechnology is a promising strategy for tumor-targeted therapy.

In the present study, a novel piRNA, hsa_piR_016975, was found to be overexpressed in HCC, especially in sorafenib-resistant HCC patients. Further studies revealed that hsa_piR_016975, acting as an oncogene, could target inhibition of the expression of serpin family B member 5 (SERPINB5; also known as Maspin) while up-regulating GPX4 expression, thereby weakening ferroptosis and resulting in HCC progression and sorafenib resistance. In addition, a novel drug delivery system was constructed in which polylactic-co-glycolic acid (PLGA) nanoparticles were packaged with sorafenib and hsa_piR_016975 inhibitor (in-016975), and coated with HCC cell membrane (yielding in-016975/Sora@PLGA-CM). It was highly efficient at reversing HCC progression and sorafenib resistance by inducing hsa_piR_016975/Maspin/GPX4 axis-mediated ferroptosis in subcutaneous xenograft and orthotopic HCC mouse models. This study highlights an appealing piRNA-target therapeutic strategy to overcome chemoresistance in HCC.

## Materials and Methods

### Human sample collection

HCC tissues and paired adjacent nontumor tissues were obtained from 56 patients who were admitted to the Affiliated Hospital of Nantong University between 2016 and 2019 and were pathologically confirmed. In our enrolled cohort, all cases did not receive any therapy before surgery. Additionally, all subjects received sorafenib treatment after surgical resection and written informed consent was obtained from all of them. The study was approved by the Ethical Committee of the Affiliated Hospital of Nantong University. The clinicopathological parameters of the patients are shown in Table S1.

### Cell culture

The HCC cell lines (HCCLM3, SK-Hep-1, PLC/PRF/5, and Huh-7) were obtained from Shanghai Cell Bank of the Chinese Academy of Sciences. The cells were grown in Dulbecco’s modified Eagle’s medium (Gibco, USA) or minimum essential medium (Gibco, USA) supplemented with 10% fetal bovine serum at 5% CO_2_, 37 °C.

### Vector construction and transfection

The hsa_piR_016975 mimics (mi-016975) and its inhibitors (in-016975) were synthesized and transfected using the RiboFECT CP Transfection Kit (RiboBio, China). Si-Maspin and its overexpressed vectors were constructed (GenePharma, China) and transfected using Lipofectamine 3000 (Invitrogen, USA).

### RNA quantification

RNA was isolated using TRIzol (Invitrogen, USA) and reverse-transcribed into cDNA using the RevertAid RT Kit (Thermo Fisher Scientific, USA). Quantitative real-time polymerase chain reaction (qRT-PCR) was performed via QuantStudio5 (Thermo Fisher Scientific, USA) using 2× Universal SYBR Green Fast qPCR Mix, and the results were analyzed by 2^−ΔΔCt^ method. U6 was applied as an endogenous control for hsa_piR_016975. 18S was used as an endogenous control for Maspin and GPX4. The primer sequences were listed in Table S2.

### Cellular proliferation assays

A cell suspension of 3000 cells/ml was inoculated into a 96-well plate (100 μl/well). To each well, 10 μl of Cell Counting Kit-8 (CCK-8, DOJINDO, Japan) was added, and the mixture was incubated for 2 h. Then, the absorbance at 450 nm was measured in a microplate reader. For 5-ethynyl-2′ -deoxyuridine (EdU) assay, Cell-Light EdU Apollo 567 In Vitro Kit was applied according to the manufacturer’s manual (RiboBio, China) and analyzed using a fluorescence microscope (Olympus, Japan).

### Cellular apoptosis assay

Approximately 5 × 10^5^ HCC cells were added with 500 μl of 1× binding buffer. Then, Annexin V-allophycocyanin (APC)/propidium iodide (PI) (MULTI SCIENCES, China) was mixed to each tube at room temperature to avoid exposure to light. Flow cytometry was used to detect fluorescent signals (BD LSRFortessa, USA).

### Transwell assay

For the migration experiment, HCC cells (5 × 10^4^/ml) were resuspended in serum-free medium and added to the upper chamber, and then the complete medium was added to the lower chamber. After 48 h of culture, the cells were stained and counted under a microscope (Olympus, Japan). For the invasion experiment, a coating of diluted Matrigel (BD Biosciences, USA) was applied to the bottom of the upper chamber and the remaining procedures were identical to the migration experiment.

### Western blot assay

Proteins were collected with the protein lysates (radioimmunoprecipitation assay and phenylmethylsulfonyl fluoride) and dissociated via sodium dodecyl sulfate–polyacrylamide gel electrophoresis transferred to polyvinylidene difluoride membranes and blocked with 5% nonfat milk. The membrane was incubated with primary antibodies, including Maspin, GPX4, N-cadherin, E-cadherin, vimentin, glyceraldehyde-3-phosphate dehydrogenase (GAPDH), and β-actin, and secondary antibodies (Proteintech, China). The bands were analyzed using a chemiluminescence kit (Thermo Fisher Scientific, USA).

### Immunoprecipitation analysis

The Pierce Co-immunoprecipitation Kit (Thermo Fisher Scientific, USA) was applied for immunoprecipitation analysis. The cell lysates were cocultivated with the antibody overnight at 4 °C for Western blot analysis.

### Cellular metabolic assays

ROS level was measured with a ROS assay kit (Beyotime, China). The GSH Assay Kit and the GSSG Assay Kit (Solarbio, China) were used to determine the cellular glutathione (GSH) and glutathione disulfide (GSSG) levels, respectively, to calculate the GSH/GSSG ratio. Cellular malondialdehyde (MDA) level was measured with the MDA assay kit for detecting lipid peroxidation (Solarbio, China). The above operations were conducted according to the manufacturer’s instructions.

### Establishment of sorafenib-resistant cell lines

HCCLM3 and PLC/PRF/5 cells in the logarithmic growth stage were cultured with 1 μM sorafenib. After 2 weeks of culture, the drug concentration was gradually increased. Finally, the IC_50_ (median inhibitory concentration) values of HCCLM3 and PLC/PRF5 were 5.4 and 4.8 μM, as the screening concentrations, respectively. The drug-resistant cells that had survived for 4 weeks were identified and transferred to proper culture plates. The sorafenib concentration was maintained during continuous culture. After 12 weeks of culture, sorafenib-resistant (HCCLM3-SR and PLC/PRF/5-SR) cells were established.

### Preparation and characterization of in-016975/Sora@PLGA-CM

PLGA nanoparticles were produced according to the water/oil/water solvent evaporation method. Thirty micrograms of in-016975 plasmid and 10 mg of sorafenib were added to 1 ml of PLGA nanoparticles. After being incubated for 20 min at room temperature, the complexes were centrifuged (1,800*g*, 20 min) to remove the unbound plasmid from the supernatant. Subsequently, in-016975/Sora@PLGA was mixed with the HCC cell membranes, which were extracted according to previous methods [19,21]. The mixture was sonicated using an Avanti mini extruder and extruded 20 times through 400- and 200-nm nuclear pore membranes to form in-016975/Sora@PLGA-CM.

### Construction of xenograft model and orthotopic HCC mouse model

For construction of xenograft model, 6-week BALB/c nude mice were randomly divided into 2 groups. In-016975/Sora@PLGA-CM- and in-NC/Sora@PLGA-CM-treated HCCLM3-SR cells (1 × 10^7^ cells) were inoculated into the left axilla of nude mice. The tumor volume was recorded on days 7, 12, 17, 22, and 27. For construction of orthotopic HCC model, the BALB/c nude mouse abdomen was dissected after being anesthetized, and then 1 × 10^6^ HCCLM3-SR cells were infused into the appropriate part of the liver. The nude mice were treated every other day with physiological saline, Sora@PLGA-CM, in-016975@PLGA-CM, or in-016975/Sora@ PLGA-CM. The experimental data were collected on days 7, 14, and 21. After the mice were sacrificed, the tumors were surgically collected and embedded in paraffin for immunohistochemical (IHC) staining. All animal experiments were approved by the Ethics Committee of the Laboratory Animal Center of Nantong University.

### Statistical analysis

The results are presented as the mean ± standard deviation and were analyzed using SPSS software. *t* Test and one-way analysis of variance (ANOVA) were applied to compare the data in the different groups. The association between hsa_piR_016975 expression and clinicopathological features was assessed using chi-square test. Kaplan−Meier analysis was performed to assess the survival rate. *P* < 0.05 was considered as statistical significance.

## Results

### Hsa_piR_016975 is highly expressed in HCC

**Fig. 1.**
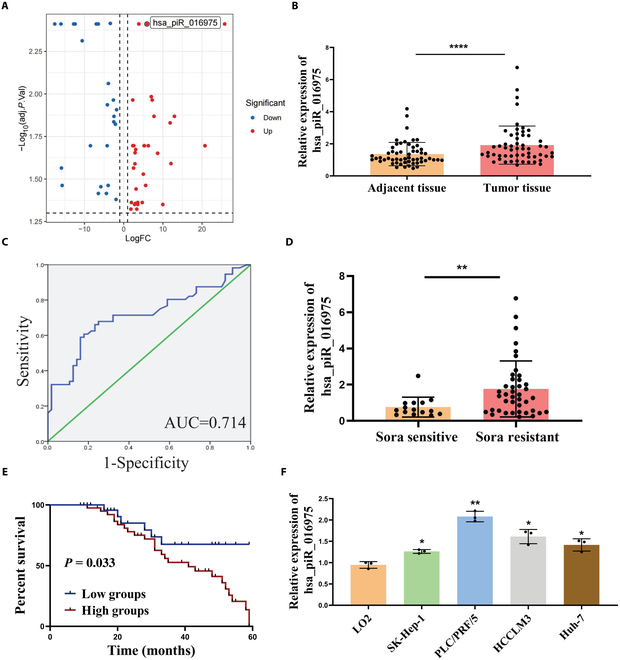
Hsa_piR_016975 is highly expressed in HCC. (A) Differentially expressed piRNAs in HCC from the BioStudies Database. (B) Detection of hsa_piR_016975 expression in HCC tissues and paired adjacent normal tissues by qRT-PCR (*n* = 56). (C) A ROC curve was used to estimate the diagnostic value of hsa_piR_016975 for HCC. (D) Analysis of the relationship between the hsa_piR_016975 expression level and sorafenib resistance in patients with HCC. (E) Survival curve analysis of the association between the hsa_piR_016975 expression level and overall survival in HCC patients. (F) Detection of hsa_piR_016975 expression in HCC cell lines (SK-Hep-1, PLC/PRF/5, Huh-7, and HCCLM3) and a normal human liver cell line (LO2) by qRT-PCR. *P < 0.05, **P < 0.01, ****P < 0.0001.

First, the BioStudies Database (E-MTAB-3973) was used to screen for dysregulated piRNAs in HCC (Fig. 1A), from which the most 4 overexpressed piRNAs (hsa_piR_016975, hsa_piR_017724, hsa_piR_001170, and hsa_piR_019951) were selected and analyzed in 20 paired HCC tissues via qRT-PCR. The results demonstrated that only hsa_piR_016975 expression was increased in HCC, whereas the expression of the other 3 piRNAs had no significant difference or exhibited an opposite pattern to that observed in the database analysis (Fig. S1A to D). The expression of hsa_piR_016975 was further verified to be markedly overexpressed in 56 HCC tissues (*P* < 0.0001; Fig. 1B). Additionally, the area under the receiver operating characteristic (ROC) curve was 0.714, suggesting that hsa_piR_016975 has potential diagnostic value for HCC (Fig. 1C). Moreover, the association analysis demonstrated that hsa_piR_016975 expression was closely related with tumor differentiation (*P* = 0.026) and tumor node metastasis (TNM) stage (*P* = 0.008) (Table S1). Interestingly, HCC patients with high levels of hsa_piR_016975 might be predisposed to sorafenib resistance (Fig. 1D). Furthermore, the survival curve indicated that HCC cases with high level of hsa_piR_016975 owned substantially shorter overall survival (Fig. 1E). In addition, hsa_piR_016975 expression was also enhanced in 4 HCC cell lines, particularly in PLC/PRF/5 and HCCLM3 cells. Similarly, Feng et al. [22] found that the IC_50_ of HCCLM3 and PLC/PRF/5 was higher than that of Huh7 cells (Fig. 1F). These results indicated that up-regulated hsa_piR_016975 may have a momentous role in hepatocarcinogenesis and sorafenib resistance.

### Hsa_piR_016975 assists HCC malignant progression in vitro

**Fig. 2.**
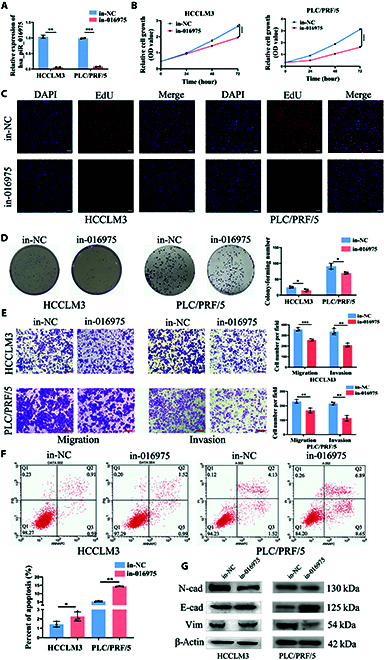
Hsa_piR_016975 assists HCC malignant progression in vitro. (A) Detection of the efficiency in HCCLM3 and PLC/PRF/5 cells transfected with hsa_piR_016975 inhibitors (in-016975) or empty vector control (in-NC). (B to D) CCK-8, EdU, and colony formation assays were performed to analyze the abilities of proliferation in in-016975- or in-NC-transfected HCC cells (scale bar, 100 μm). (E) The migration and invasion abilities were detected by transwell assays in in-016975- or in-NC-transfected HCC cells (scale bar, 100 μm). (F) Flow cytometry was used to analyze the cell apoptosis in in-016975- or in-NC-transfected HCC cells. (G) Western blot analyzed the expressions of N-cadherin, E-cadherin, and vimentin in in-016975- or in-NC-transfected HCC cells. *P < 0.05, **P < 0.01, ***P < 0.001, ****P < 0.0001.

To identify the biological functions of hsa_piR_016975, HCCLM3 and PLC/PRF/5 cells were treated with hsa_piR_016975 inhibitor (in-016975), whereas SK-Hep-1 cells were treated with hsa_piR_016975 mimics (mi-016975). According to the results of transfection efficiencies, both in-016975 and mi-016975 were swimmingly transfected into 3 HCC cell lines (Fig. 2A and Fig. S2A). In vitro experiments displayed that abilities of cell proliferation (CCK-8, EdU, and colony formation assays) and metastasis (cell migration and invasion assays) were strongly inhibited in in-016975-treated HCCLM3 and PLC/PRF/5 cells. However, in mi-016975-treated SK-Hep-1 cells, the opposite trend was discovered (Fig. 2B to E and Fig. S2B to E). Additionally, the apoptotic rate was obviously raised in in-016975-treated HCCLM3 and PLC/PRF/5 cells, whereas it was decreased in mi-016975-treated SK-Hep-1 cells (Fig. 2F and Fig. S2F). Also, the expressions of epithelial mesenchymal transition (EMT) markers were examined in HCC cells. Unsurprisingly, E-cadherin was obviously up-regulated, yet vimentin and N-cadherin were overtly down-regulated in in-016975-treated HCCLM3 and PLC/PRF/5 cells. Nevertheless, the opposite EMT trend was detected in mi-016975-treated SK-Hep-1 cells (Fig. 2G and Fig. S2G). Collectively, hsa_piR_016975 facilitates HCC cell growth, metastasis, and EMT formation in vitro*.*

### Hsa_piR_016975 promotes HCC progression by targeting inhibition of Maspin

**Fig. 3.**
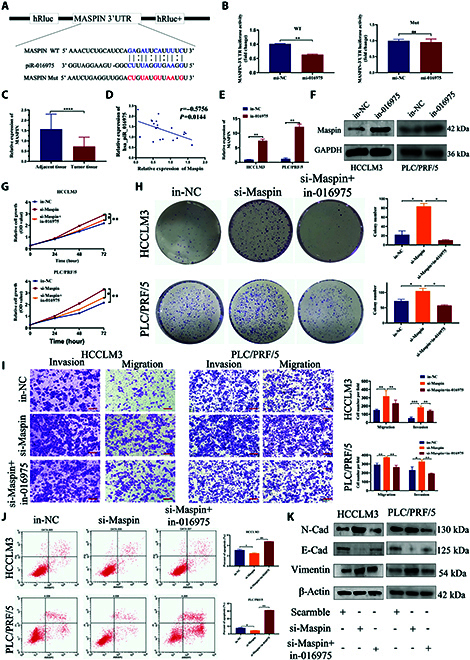
Hsa_piR_016975 promotes HCC progression by targeting inhibition of Maspin. (A) Diagram of the binding sites of piRNA-016975 to the 3′-UTR of Maspin mRNA. (B) Dual-luciferase analysis of the luciferase activity in HCC cells cotreated with mi-016975 and wild-type (WT) or mutation (Mut) vectors of Maspin 3′-UTR mRNA. (C) Detection of Maspin expression in HCC tissues by qRT-PCR. (D) The relationship between Maspin and piRNA-016975 expressions in HCC was analyzed using Pearson correlation analysis. (E and F) The mRNA and protein expression levels of Maspin were analyzed in in-NC- or in-016975-treated HCCLM3 and PLC/PRF/5 cells. (G and H) CCK-8 and colony formation assays were performed in in-NC-, si-Maspin-, or si-Maspin + in-016975-transfected HCC cells. (I) The migration and invasion abilities were detected in in-NC-, si-Maspin-, or si-Maspin + in-016975-transfected HCC cells (scale bar, 100 μm). (J) Flow cytometry was used to analyze the cell apoptosis in in-NC-, si-Maspin-, or si-Maspin + in-016975-transfected HCC cells. (K) Western blot analysis of the expressions of N-cadherin, E-cadherin, and vimentin in in-NC-, si-Maspin-, or si-Maspin + in-016975-transfected HCC cells. *P < 0.05, **P < 0.01, ***P < 0.001, ****P < 0.0001. ns, not significant.

To investigate piR-016975’s regulatory mechanisms in HCC, nucleoplasmic separation assay unlocked that piR-016975 was primarily located in the cytoplasm (Fig. S3A). Research has shown that piRNAs in the cytoplasm target the transposon sequences in the 3′-untranslated region (3′-UTR) of mRNA, causing its degradation [23,24]. The miRDB (https://mirdb.org/) and targetScan databases (http://www.targetscan.org/vert_72/), as well as RNAhybrid tool (https://bibiserv.cebitec.uni-bielefeld.de) were subsequently used to predict target genes of hsa_piR_016975. Then, 4 potential targets (Maspin, UBE2A, APLP2, and PLRG1) to hsa_piR_016975 were screened. The RNA-Protein Interaction Prediction (RPISeq) database (http://pridb.gdcb.iastate.edu/RPISeq/) further demonstrated that Maspin had a higher binding affinity for hsa_piR_016975 than the other 3 target genes (Fig. S3B and C). Moreover, hsa_piR_016975 had potential binding sites in the 3′-UTR of Maspin mRNA and dual-luciferase analysis further validated that Maspin was a direct target gene of hsa_piR_016975 (Fig. 3A and B). Maspin is a member of the serine protease inhibitor superfamily that represses the progression of many tumors, including liver cancer [25–27]. We also found that Maspin was lowly expressed in HCC tissues and had a negative correlation with piR-016975 expression (*r* = −0.5756, *P* = 0.0144; Fig. 3C and D). Meanwhile, in in-016975-treated HCC cells, the mRNA and protein expressions of Maspin were correspondingly elevated, yet they were noticeably dropped in mi-016975-treated ones (Fig. 3E and F and Fig. S4A and B), suggesting that piR-016975 could negatively regulate the expression of its target, Maspin.

To demonstrate whether piR-016975 affected HCC growth and metastasis by targeting Maspin, RNA interference vector targeting Maspin (si-Maspin) and Maspin overexpressed vector (pc-Maspin) were constructed to implement rescue experiments (Fig. S4C and D). Furthermore, we also found that overexpression of Maspin could cause changes in the expression of GPX4 (Fig. S4E and F). According to the CCK-8, colony formation, and Transwell assays, in-016975 toppled the facilitating effects on cell proliferation, migration, and invasion in si-Maspin cotreated HCCLM3 and PLC/PRF/5 cells, while mi-016975 could partially recover the inhibitory effects on cell proliferation, migration, and invasion in pc-Maspin cotreated SK-Hep-1 cells (Fig. 3G to I and Fig. S5A to C). Moreover, flow cytometry showed that in-016975 reversed the reduction of apoptosis rate in si-Maspin cotreated HCCLM3 and PLC/PRF/5 cells, whereas mi-016975 overturned the increase of apoptosis rate in pc-Maspin cotreated SK-Hep-1 cells (Fig. 3J and Fig. S5D). Studies have shown that Maspin can decrease cell invasion and migration of malignant tumors by inhibiting EMT [28,29]. Our western blot analysis demonstrated that in-016975 could subvert the inhibitory effects on E-cadherin expression and the promoting effects on N-cadherin and vimentin expression in si-Maspin cotreated HCCLM3 and PLC/PRF/5 cells, yet mi-016975 could overthrow the facilitating effect on E-cadherin expression and the inhibitory effects on N-cadherin and vimentin expression in pc-Maspin cotreated SK-Hep-1 cells (Fig. 3K and Fig. S5E). These data suggests that piR-016975 boosts HCC growth, metastasis, and EMT formation by targeting inhibition of Maspin expression.

### Hsa_piR_016975 induces sorafenib resistance by inhibiting ferroptosis in HCC

**Fig. 4.**
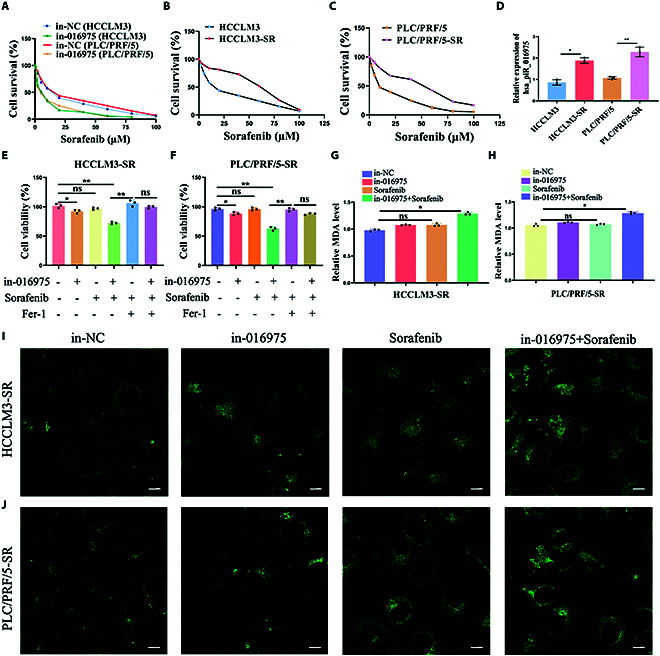
Hsa_piR_016975 induces sorafenib resistance by inhibiting ferroptosis- associated lipid peroxidation in HCC. (A) Increasing doses of sorafenib were used to treat in-NC- or in-016975-transfected HCCLM3 and PLC/PRF/5 cells, and the corresponding IC_50_ was determined from the dose–response curve. (B and C) Primary cell lines (HCCLM3 and PLC/PRF/5) and sorafenib-resistant cell lines (HCCLM3-SR and PLC/PRF/5-SR) were treated with increased doses of sorafenib, and the corresponding IC_50_ was determined from the dose–response curve. (D) Detection of hsa_piR_016975 expression in sorafenib-resistant cell lines by qRT-PCR. (E and F) Cell viability was analyzed with a CCK-8 kit in HCCLM3-SR and PLC/PRF/5-SR cells cotreated with in-016975, sorafenib, or Fer-1. (G and H) Cellular MDA levels in in-016975, sorafenib, or in-016975 + sorafenib cotreated HCCLM3-SR and PLC/PRF/5-SR cells were determined with an MDA assay kit. (I and J) LPO deposition in in-016975, sorafenib, or in-016975 + sorafenib cotreated HCCLM3-SR and PLC/PRF/5-SR cells was observed via fluorescence microscopy (scale bar, 10 μm). *P < 0.05, **P < 0.01.

As shown in Fig. 1D, hsa_piR_016975 had a higher up-regulation in sorafenib-resistant HCC patients. Subsequently, sorafenib was used to treat in-016975-transfected HCC cells. Interestingly, the IC_50_ values were decreased in both in-016975-treated HCCLM3 and PLC/PRF/5 cells, indicating that hsa_piR_016975 could stimulate sorafenib resistance in HCC (Fig. 4A). Moreover, sorafenib-resistant HCC cell lines (HCCLM3-SR and PLC/PRF/5-SR) were generated via an intermittent dose escalation strategy [30,31]. As compared with HCCLM3 and PLC/PRF/5 cells, the IC_50_ values of both HCCLM3-SR and PLC/PRF/5-SR cells were significantly raised after treatment with different concentrations of sorafenib (Fig. 4B and C). Moreover, piR-016975 expression in HCCLM3-SR and PLC/PRF/5-SR cell was higher than that in HCCLM3 and PLC/PRF/5 cells (Fig. 4D). Recent studies have shown that ferroptosis is caused by LPO product accumulation, glutathione (GSH) synthesis, and iron overload and mediates sorafenib resistance in HCC patients [15,32,33]. Hence, we speculated that ferroptosis might be involved in hsa_piR_016975-induced sorafenib resistance. As expected, our cell viability assay showed that cell proliferation was inhibited in the sorafenib + in-016975 cotreated HCCLM3-SR and PLC/PRF/5-SR cells, yet the ferroptosis inhibitor ferrostatin-1 (Fer-1) effectively reversed this inhibitory effect, revealing that ferroptosis was actually involved in hsa_piR_016975-induced sorafenib resistance (Fig. 4E and F).

**Fig. 5.**
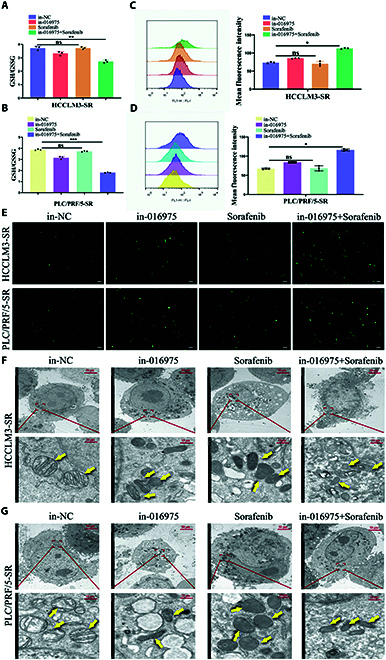
Hsa_piR_016975 induces sorafenib resistance by inhibiting ferroptosis- associated ROS accumulation in HCC. (A and B) The GSH Assay Kit and GSSG Assay Kit were used to determine the cellular GSH and GSSG levels in in-016975, sorafenib, or in-016975 + sorafenib cotreated HCCLM3-SR and PLC/PRF/5-SR cells. (C and D) ROS accumulation in in-016975, sorafenib, or in-016975 + sorafenib cotreated HCCLM3-SR and PLC/PRF/5-SR cells was detected by the fluorescence probe dichlorodihydrofluorescein diacetate (DCFH-DA) via flow cytometry. (E) ROS accumulation in in-016975, sorafenib, or in-016975 + sorafenib cotreated HCCLM3-SR and PLC/PRF/5-SR cells was detected by the fluorescence probe DCFH-DA via fluorescence microscope (scale bar, 100 μm). (F and G) The mitochondrial morphology in in-016975, sorafenib, or in-016975 + sorafenib cotreated HCCLM3-SR and PLC/PRF/5-SR cells was observed via transmission electron microscopy (TEM). *P < 0.05, **P < 0.01, ***P < 0.001.

Additionally, ferroptosis-related biomarkers, such as the GSH/GSSG ratio, MDA and ROS levels, and mitochondrial morphology, were further analyzed. The MDA levels were prominently higher in in-016975 + sorafenib cotreated HCCLM3-SR and PLC/PRF/5-SR cells than in-NC, in-016975, and in-NC + sorafenib treatment groups (Fig. 4G and H). Additionally, the degree of lipid peroxidation in in-016975 + sorafenib cotreated HCCLM3-SR and PLC/PRF/5-SR cells was significantly increased, as determined via fluorescence microscopy, revealing that in-016975 could enhance lipid deposition in sorafenib-resistant HCC cells (Fig. 4I and J). Besides, the GSH/GSSG ratio was markedly lower and the ROS level was prominently higher in in-016975 + sorafenib cotreated HCCLM3-SR and PLC/PRF/5-SR cells than in-NC, in-016975, and in-NC + sorafenib treatment groups, suggesting that in-016975 could heighten the oxidative stress response in sorafenib-resistant HCC cells (Fig. 5A to E). Furthermore, the damage degree of mitochondrial morphology in in-016975 + sorafenib cotreated HCCLM3-SR and PLC/PRF/5-SR cells was markedly strengthened according to transmission electron microscopy (Fig. 5F and G). Collectively, these results uncover that hsa_piR_016975 induces sorafenib resistance by impeding ferroptosis in HCC cells.

### Inhibition of hsa_piR_016975 enhances the sensitivity of HCC cells to sorafenib by activating Maspin/GPX4-mediated ferroptosis

**Fig. 6.**
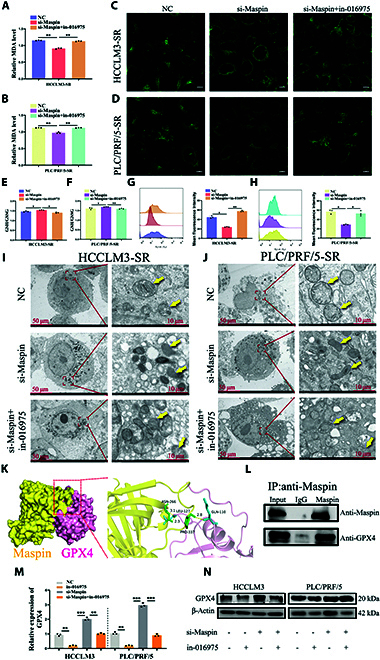
Inhibition of hsa_piR_016975 enhances the sensitivity of HCC cells to sorafenib by activating Maspin/GPX4-mediated ferroptosis. (A and B) Cellular MDA levels in si-Maspin or si-Maspin + in-016975 cotreated HCCLM3-SR and PLC/PRF/5-SR cells were determined using an MDA assay kit between the NC group. (C and D) LPO deposition in si-Maspin or si-Maspin + in-016975 cotreated HCCLM3-SR and PLC/PRF/5-SR cells was observed via fluorescence microscopy (scale bar, 10 μm). (E and F) The GSH Assay Kit and GSSG Assay Kit were used to determine the cellular GSH and GSSG levels in si-Maspin or si-Maspin + in-016975 cotreated HCCLM3-SR and PLC/PRF/5-SR cells. (G and H) ROS accumulation was detected by the fluorescence probe DCFH-DA via flow cytometry in si-Maspin or si-Maspin + in-016975 cotreated HCCLM3-SR and PLC/PRF/5-SR cells. (I and J) The mitochondrial morphology in si-Maspin or si-Maspin + in-016975 cotreated HCCLM3-SR and PLC/PRF/5-SR cells was observed via TEM. (K) Molecular docking analysis of the binding sites between Maspin and GPX4. (L) The interaction between GPX4 and Maspin was verified by coimmunoprecipitation assay. (M and N) qRT-PCR and Western blot analyses were applied to detect GPX4 mRNA and protein expression in in-016975, si-Maspin, or si-Maspin + in-016975 cotreated HCCLM3 and PLC/PRF/5 cells. *P < 0.05, **P < 0.01.

As a target gene to hsa_piR_016975, whether Maspin took part in the process of ferroptosis in sorafenib-resistant cells was further explored. Our cell viability assay showed that cell proliferation was inhibited in pc-Maspin-treated HCCLM3-SR and PLC/PRF/5-SR cells; however, Fer-1 effectively reversed this inhibitory effect, demonstrating that Maspin was actually involved in the process of ferroptosis in sorafenib-resistant cells (Fig. S6A and B). Then, the rescue experiments to analyze ferroptosis-related biomarkers were further performed. The results unmasked that in-016975 could reverse the inhibitory effects on MDA level, the degree of lipid peroxidation, ROS level, and the degree of mitochondrial morphology damage, while in-016975 could overturn the promoting effects on the GSH/GSSG ratio in si-Maspin cotreated HCCLM3 and PLC/PRF/5 cells (Fig. 6A to J). These data suggest that Maspin is involved in hsa_piR_016975-induced sorafenib resistance by regulating ferroptosis in HCC.

Based on the bioinformatic prediction, we found potential interaction between Maspin and 4 ferroptosis-associated proteins (GPX4, ACSL4, TFRC, and SLC7A11). Subsequently, the Gene Expression Profiling Interactive Analysis (GEPIA, http://gepia.cancer-pku.cn/) database was applied to analyze the correlation between Mapin and GPX4, ACSL4, TFRC, and SLC7A11 expression levels, and the results showed that rather than the other 3, only Maspin was significantly negatively correlated with GPX4 (Fig. S7A to E). Furthermore, we detected the mRNA levels of GPX4, ACSL4, TFRC, and SLC7A11 in HCCLM3-SR and PLC/PRF/5-SR cells. The results showed that in-016975 could also significantly inhibit the levels of GPX4 (Fig. S8A and B). GPX4, as a crucial regulator of ferroptosis, reduces lethal LPOs to inhibit ferroptosis. Its expression was decreased in sorafenib treatment cells, suggesting the induction of ferroptosis [34]. Notably, GPX4 expression was dramatically higher in HCC tissues than in adjacent normal tissues, and correlation analysis showed that it was negatively correlated with Maspin expression, while it was positively correlated with hsa_piR_016975 expression (Fig. S9A to C). The existence of interaction sites between Maspin and GPX4 was predicted by using UniProt (https://www.uniprot.org/), HDOCK software, and Pymol software (Fig. 6K). Subsequently, the interaction between Maspin and GPX4 was further confirmed by using a coimmunoprecipitation assay (Fig. 6I). Furthermore, qRT-PCR and Western blot analyses revealed that si-Maspin could up-regulate GPX4 mRNA and protein expressions in both HCCLM3 and PLC/PRF/5 cells, while si-Maspin + in-016975 could down-regulate the expressions of GPX4, showing that hsa_piR_016975 regulated GPX4 expression by targeting Maspin in HCC cells (Fig. 6M and N). In summary, these results manifest that in-016975 heightens the sensitivity of HCC cells to sorafenib by inducing Maspin/GPX4-mediated ferroptosis.

### Synthesis and characterization of the nanocomposites in-016975/Sora@PLGA-CM

**Fig. 7.**
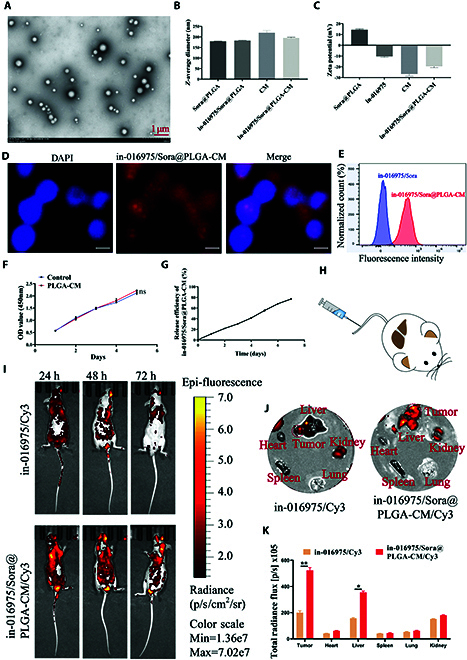
Preparation and characterization of in-016975/Sora@PLGA-CM (scale bar, 1 μm). (A) Visualization of in-016975/Sora@PLGA-CM via TEM. (B) Analysis of the *Z*-average diameter of in-016975/Sora@PLGA-CM. (C) Analysis of the zeta potentials of in-016975/Sora@PLGA-CM. (D and E) Fluorescence microscopy and flow cytometry were used to detect the degree of absorption of in-016975/Sora@PLGA-CM in HCC cells (scale bar, 10 μm). (F) In vitro cytotoxicity of PLGA-CM nanoparticles to HCC cells and the untreated HCC cells as the control group. (G) In vitro release profiles of in-016975 from in-016975/Sora@PLGA-CM and RNA concentration were calculated by measuring the absorption at 260 nm with an ultraviolet spectrophotometer. (H and I) Representative images of the changes in fluorescence intensity between mice with the injection of in-016975/Sora@PLGA-CM/Cy3 or in-016975/Cy3 into the tail vein in vivo. (J and K) Biodistribution of in-016975/Sora@PLGA-CM/Cy3 or in-016975/Cy3 in major organs of the mice, such as the liver, heart, kidney, spleen, and lung. *P < 0.05, **P < 0.01.

To assess the treatment effects of in-016975 on the sorafenib-resistant HCC, we designed and synthesized in-016975/Sora@PLGA-CM nanoparticles. First, polyethyleneimine (PEI) was used to modify PLGA to obtain a positive surface charge. Hence, in-016975 and sorafenib could rely on their negative surface charge to bind to PLGA, thereby forming in-016975/Sora@PLGA. Then, the HCC cell membrane and in-016975/Sora@PLGA were mixed by sonication and repeated extrusion. Transmission electron microscopy clearly showed that in-016975/Sora@PLGA coated the cell membrane (Fig. 7A). Moreover, the average hydrodynamic diameter of in-016975/Sora@PLGA-CM was approximately 190 nm, and its zeta potential was approximately −18 mV (Fig. 7B and C). In addition, after coculture of in-016975/Sora@PLGA-CM with HCC cells, both fluorescence microscopy and flow cytometry analyses showed that the vast majority of in-016975/Sora@PLGA-CM was absorbed in the HCC cells (Fig. 7D and E). Furthermore, the CCK-8 assay confirmed that the PLGA-CM nanoparticles did not induce significant cytotoxicity and did not impact the proliferation of HCC cells (Fig. 7F). Additionally, an in vitro release assay revealed that encapsulation of the cell membrane did not affect in-016975 release, and approximately 70% of the loaded in-016975 was released within 1 week (Fig. 7G). Additionally, we tested the targeting ability of in-016975/Sora@PLGA-CM in vivo orthotopic HCC mouse models. Fluorescence imaging indicated that the nanocomposites were enriched in the tumor site of the mice. In particular, there was still a strong fluorescence signal at the tumor site after 72 h of treatment with in-016975/Sora@PLGA-CM/Cy3, and this signal was significantly stronger than in the in-016975/Cy3 treatment group. In addition, weak fluorescence signals were always observed in the kidney, heart, spleen, and lung in both treatment groups (Fig. 7H to K). Taken together, these results suggested that in-016975/Sora@PLGA-CM was successfully constructed and had good efficacy, safety, and continuous targeting to tumor cells.

### In-016975/Sora@PLGA-CM suppresses sorafenib-resistant HCC progression in the subcutaneous xenograft model

To investigate the therapeutic efficacy of in-016975/Sora@PLGA-CM in vivo, in-016975/Sora@PLGA-CM-treated HCCLM3-SR cells were inoculated subcutaneously into nude mice. The growth curve indicated that tumor growth was visibly slower in the in-016975/Sora@PLGA-CM group than in the in-NC/Sora@PLGA-CM group. Twenty-seven days after treatment, the tumors were substantially smaller and lighter in the in-016975/Sora@PLGA-CM group than in the in-NC/Sora@PLGA-CM group (Fig. S10A to D). Moreover, the expression of hsa_piR_016975 also decreased in in-016975/Sora@PLGA-CM-treated transplanted tumors (Fig. S10E). In addition, IHC staining revealed that the percentages of Ki67- and Bcl2-positive cells were obviously declined in the in-016975/Sora@ PLGA-CM group. Moreover, E-cadherin expression was markedly increased, yet vimentin expression was noticeably reduced in the in-016975/Sora@PLGA-CM group (Fig. S10F). These results suggest that in-016975/Sora@PLGA-CM restrains the malignant progression of sorafenib-resistant HCC cells in vivo.

### In-016975/Sora@PLGA-CM arrests sorafenib-resistant HCC progression in the orthotopic transplantation model

**Fig. 8.**
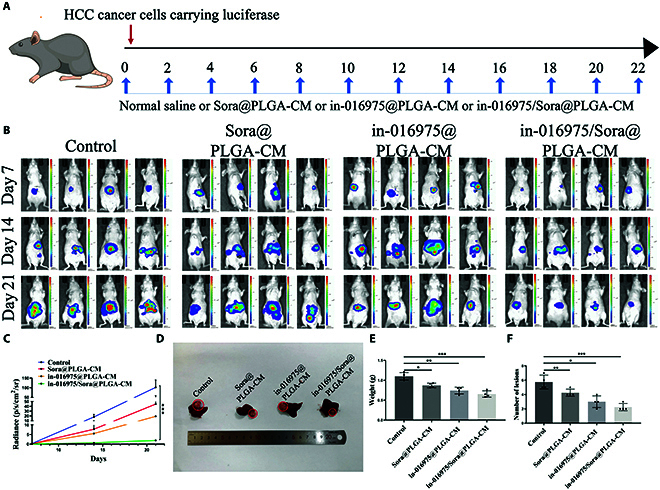
In-016975/Sora@PLGA-CM inhibits the growth of sorafenib-resistant HCC in the orthotopic transplantation model. (A) Schematic illustration of the treatment regimen for orthotopic HCC model. (B) Representative images of fluorescence intensity in orthotopic HCC mice inoculated with physiological saline (control), Sora@PLGA-CM, in-016975@PLGA-CM, or in-016975/Sora@PLGA-CM-treated HCCLM3-SR cells. (C to F) Fluorescence intensity alterations, tumor volume, tumor weights, and lesion numbers were detected in 4 groups. *P < 0.05, **P < 0.01, ***P < 0.001.

To further investigate the treatment effects of in-016975/Sora@PLGA-CM on sorafenib-resistant HCC in vivo, orthotopic HCCLM3-SR mouse models were established. Physiological saline, Sora@PLGA-CM, in-016975@PLGA-CM, and in-016975/Sora@PLGA-CM were injected into HCCLM3-SR model mice every 2 d for 22 d (Fig. 8A). The fluorescence intensity of the models was detected via fluorescence imaging in vivo. Mice treated with Sora@PLGA-CM or in-016975@PLGA-CM demonstrated a weaker fluorescent signal compared to those in the control group. Moreover, the fluorescent signal intensity was further decreased in the in-016975/Sora@PLGA-CM treatment group. On the 7th, 14th, and 21st days, the in-016975/Sora@PLGA-CM treatment group had the lowest fluorescence intensity (Fig. 8B and C). Additionally, the in-016975/Sora@PLGA-CM treatment group had the smallest tumor volume, weight, and the fewest lesions (Fig. 8D to F). These findings indicate that in-016975 combined with sorafenib hinders the growth of orthotopic HCC and makes tumors more sensitive to sorafenib.

**Fig. 9.**
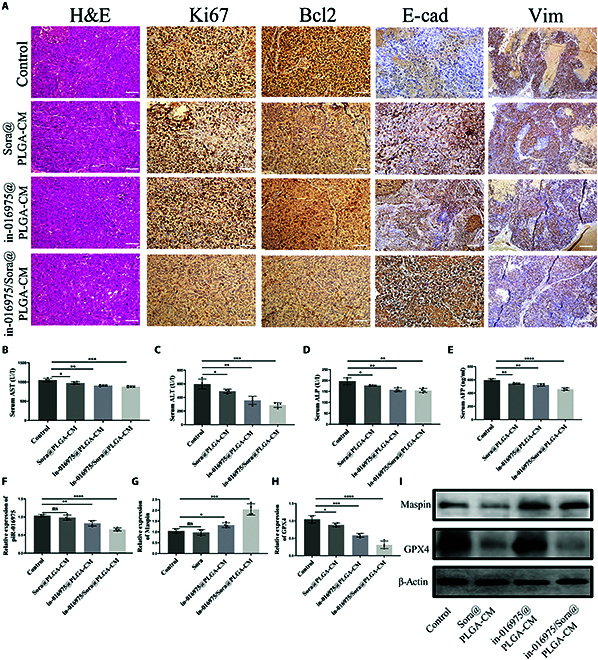
In-016975/Sora@PLGA-CM represses HCC progression and sorafenib resistance by inducing hsa_piR_016975/Maspin/GPX4 axis-mediated ferroptosis in the orthotopic HCC model. (A) Representative hematoxylin and eosin (H&E) staining images and IHC images of Ki67, Bcl2, E-cadherin, and vimentin in HCC orthotopic mice inoculated with physiological saline (control), Sora@PLGA-CM, in-016975@PLGA-CM, or in-016975/Sora@PLGA-CM-treated HCCLM3-SR cells (scale bar, 50 μm). (B to E) Common clinical liver function indices, such as serum AST, ALT, ALP, and AFP levels, were detected in 4 groups. (F to H) qRT-PCR was used to detect the expression levels of hsa_piR_016975, Maspin, and GPX4 mRNA in 4 groups. (I) Western blot assay was used to determine the protein expression of Maspin and GPX4 in 4 groups. *P < 0.05, **P < 0.01, ***P < 0.001, ****P < 0.0001.

In addition, as compared with the Sora@PLGA-CM and in-016975@PLGA-CM treatment groups, IHC assay revealed that tumor cell proliferation was sensibly lowered and that EMT was suppressed, whereas cell apoptosis was dramatically augmented in the in-016975/Sora@PLGA-CM treatment group (Fig. 9A). Additionally, common clinical liver function indices were detected in the blood of the mice. The serum aspartate aminotransferase (AST), alanine aminotransferase (ALT), alkaline phosphatase (ALP), and α fetoprotein (AFP) levels were much lower in the in-016975/Sora@PLGA-CM treatment group than in the Sora@PLGA-CM and in-016975@PLGA-CM treatment groups, revealing its notable therapeutic effects on HCC (Fig. 9B to E). In addition, qRT-PCR revealed that hsa_piR_016975 and GPX4 mRNA expression levels were lower, whereas Maspin mRNA expression level was higher in the in-016975/Sora@PLGA-CM treatment group than in the Sora@PLGA-CM and in-016975@PLGA-CM treatment groups (Fig. 9F to H). Moreover, Western blot analyses further unveiled that GPX4 expression was prominently declined, yet Maspin expression was added in the in-016975/Sora@PLGA-CM treatment group (Fig. 9I). Altogether, the results manifest that in-016975/Sora@PLGA-CM represses HCC progression and sorafenib resistance by inducing hsa_piR_016975/Maspin/GPX4 axis-mediated ferroptosis.

## Discussion

Accumulating studies have shown that aberrantly expressed piRNAs take part in the progression of various tumors, including breast cancer, colorectal cancer, and HCC [35,36]. Some of these piRNAs act as oncogenes that can promote cancer progression. For example, piR-31106 promotes breast carcinogenesis by encouraging METTL3-mediated m6A methylation [37]. PiR-651 accelerates breast cancer cell proliferation and migration by inducing PTEN promoter methylation [38]. Nevertheless, other piRNAs play roles as tumor inhibitors that can suppress cancer progression. For example, piR-39980 prevents oncogenesis through the repression of FDFT1 expression in tongue squamous cell carcinoma [39]. PiR-017061 arrests pancreatic cancer growth by down-regulating EFNA5 expression [40]. PiR-017724 suppresses HCC development by silencing PLIN3 [41]. In general, exploring the potential mechanisms of piRNAs in the development of disease have important clinical application prospects.

In this study, we screened and verified that hsa_piR_016975 was highly expressed in HCC, which was closely associated with tumor differentiation, TNM stage, and prognosis of HCC patients. Moreover, our functional experiments demonstrated that in-016975 could suppress HCC cell proliferation, metastasis, and EMT formation and accelerate cell apoptosis. However, mi-016975 could promote HCC cell proliferation, metastasis, and EMT formation and arrest cell apoptosis. For the post-transcriptional regulation, mature piRNAs have a miRNA-like function, which can induce mRNA degradation and thus repress translation [23]. Here, we revealed that piR-016975, as a tumor-promotive piRNA, suppressed Maspin mRNA expression by binding to Maspin mRNA 3′-UTR, which in turn repressed its protein synthesis. Maspin has an inhibitory role in esophageal cancer by suppressing epidermal growth factor (EGF)-induced EMT [28]. Moreover, Maspin restrains cellular invasion and migration by preventing EMT and angiogenesis through ITGB1/FAK in gastric cancer [29]. Our rescue experiments showed that up-regulation or down-regulation of piR-016975 expression subverted the effects of Maspin on HCC cell growth, metastasis, apoptosis, and EMT formation. Therefore, these data confirm that piR-016975 promotes hepatocarcinogenesis and EMT phenotype by targeting Maspin.

Sorafenib, also known as Nexavar, can repress the proliferation and angiogenesis and stimulate apoptosis of HCC cells via multiple signaling pathways and has exhibited good therapeutic effects on extending the survival time of patients over the past decade. However, clinical practice has shown that only approximately one-third of HCC patients benefit from sorafenib therapy, and the remaining patients typically suffer from drug resistance within 6 months [42]. Hence, novel therapeutic strategies to overcome sorafenib resistance are urgently needed. In the present study, we first analyzed 56 HCC cases who received sorafenib treatment after surgical resection and found that patients with high levels of hsa_piR_016975 were prone to sorafenib resistance. Then, sorafenib was used to treat in-016975-transfected HCC cells, and the IC_50_ values showed decreasing trends, revealing that hsa_piR_016975 could promote sorafenib resistance in HCC. Mounting evidence has indicated that combination therapy with sorafenib may generate superior therapeutic results in HCC [43]. Given this, 2 sorafenib-resistant HCC cell lines, HCCLM3-SR and PLC/PRF/5-SR, were cultivated. The functional experiments revealed that in-016975 + sorafenib, rather than sorafenib treatment alone, could effectively hinder cell proliferation in 2 sorafenib-resistant HCC cell lines, indicating that targeted inhibition of hsa_piR_016975 combined with sorafenib might be an excellent strategy for treating sorafenib-resistant HCC.

Based on the above observations, the potential mechanisms through which in-016975 could increase the therapeutic responsiveness of sorafenib was further investigated. Evidence has shown that sorafenib resistance is closely associated with ferroptosis in HCC, and several signaling pathways, including Wnt/β-catenin/ferroptosis axis, Hippo/YAP signaling axis, LIFR/NF-κB/LCN2 pathway, and Nrf2/HO-1/GPX4 axis, are also involved, revealing the complicated mechanisms of sorafenib-associated ferroptosis suppression in HCC [32,44,45]. Herein, the ferroptosis inhibitor Fer-1 effectively attenuated proliferation inhibition induced by sorafenib + in-016975 cotreated sorafenib-resistant HCC cells, which verified that ferroptosis was involved in hsa_piR_016975-induced sorafenib resistance. Furthermore, ferroptosis-related biomarkers, such as the MDA and ROS levels, GSH/GSSG ratio, the degree of lipid peroxidation, and the damage degree of mitochondrial morphology, were correspondingly altered in in-016975 + sorafenib cotreated sorafenib-resistant HCC cells, revealing that the combination of in-016975 with sorafenib could induce ferroptosis in drug-resistant HCC cells.

Our results further found that as a target gene to hsa_piR_016975, Maspin also participated in hsa_piR_016975-induced sorafenib resistance. Then, the rescue experiments demonstrated that ferroptosis-related biomarkers, such as the MDA and ROS levels, the GSH/GSSG ratio, the degree of lipid peroxidation, and the damage degree of mitochondrial morphology, were reversed in in-016975 + si-Maspin cotreated sorafenib-resistant HCC cells, unveiling that Maspin was actually involved in hsa_piR_016975-induced sorafenib resistance by regulating ferroptosis in HCC. Furthermore, our bioinformatics predicted a potential regulatory relationship between Maspin- and ferroptosis-related genes, including GPX4, ACSL4, TFRC, and SLC7A11. Among them, GPX4 was further confirmed in the presence of interaction binding sites with Maspin and had a closely negative correlation with Maspin expression. In addition, si-Maspin up-regulated GPX4 expression, while si-Maspin + in-016975 down-regulated GPX4 expression in sorafenib-resistant HCC cells, disclosing that hsa_piR_016975 regulated GPX4 expression by targeting Maspin. Taken together, these findings reveal that in-016975 strengthens the HCC cells’ sensitivity to sorafenib by activating Maspin/GPX4-mediated ferroptosis.

Nanoparticle-based drug delivery systems have been demonstrated to have broad application value in tumor-targeted therapy. Nevertheless, studies have indicated that most of the delivery vehicles still exhibit low efficiency, rapid immune clearance, lack of biocompatibility, poor safety, and insufficient targeting [46–49]. Recently, tremendous success has been achieved by using cancer cell membrane-coated nanoparticles as tumor-targeting therapeutic agents. These modified nanoparticles have congenetic antigens, receptors, and proteins on the tumor cell membrane and exhibit good biocompatibility, homologous targeting ability, and immune escape ability [50]. To assess the effect of in-016975 on the treatment of sorafenib resistance in vivo, a PLGA-CM-based delivery system was developed for this study. We modified PLGA nanoparticles using HCC cell membranes, which achieved homologous targeting to tumor cells. A series of characterization experiments demonstrated that the nanocomposites of in-016975/Sora@PLGA-CM were successfully constructed. It could stably transport in-016975 and sorafenib, and had high efficiency, safety, and the ability to continuously target to tumor cells. Subcutaneous xenograft model was established and treated with in-016975/Sora@PLGA-CM. The results demonstrated that in-016975/Sora@PLGA-CM noticeably suppressed the growth, metastasis, and EMT process of sorafenib-resistant cells. Furthermore, we established orthotopic HCCLM3-SR mouse models and treated them with in-016975/Sora@PLGA-CM. The results showed that in-016975/Sora@PLGA-CM boosted the sensitivity of orthotopic HCC to sorafenib and effectively suppress tumor progression by inducing hsa_piR_016975/Maspin/GPX4 axis-mediated ferroptosis. This study, for the first time, combines piRNA inhibitor with sorafenib for the treatment of HCC. This innovative therapeutic approach ensures the maximum effects on molecular targeted therapy and has important clinical application prospects.

**Fig. 10.**
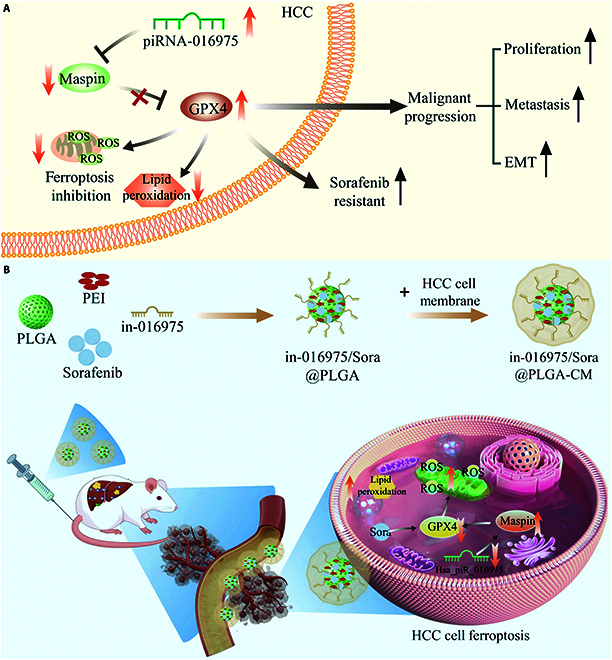
Schematic illustration of the role and mechanism of hsa_piR_016975 in HCC (A) and combination therapeutic options to overcome sorafenib resistance in HCC (B).

In summary, this study unmasked a novel molecular mechanism by which hsa_piR_016975 was involved in the malignant development of HCC, as well as an attractive combination therapy scheme for overcoming sorafenib resistance in HCC. Mechanistically, hsa_piR_016975 inhibits ferroptosis by targeting inhibition of Maspin and promoting GPX4 expression, thereby accelerating HCC progression and drug resistance (Fig. 10A). Therapeutically, HCC cell membrane-coated nanoparticles carrying sorafenib and hsa_piR_016975 inhibitor could accurately target HCC cells by activating Maspin/GPX4-mediated ferroptosis and thus increase the sensitivity of HCC cells to sorafenib (Fig. 10B). Collectively, our work proposes a new paradigm for the prospective use of piRNA-targeted treatment for sorafenib-resistant HCC.

## Data Availability

Data will be made available on request.
