# Supplementary material for: Hsa_piR_016975 Is a Novel Target of Nanotherapy that Boosts Hepatoma Progression and Sorafenib Resistance by Abating Maspin/GPX4-Mediated Ferroptosis
Source: Biomater Res. 2025 Jul 2;29:0225. doi: 10.34133/bmr.0225 (PMC12218766; doi:10.34133/bmr.0225)
Supplement: Supplementary 1 — Figs. S1 to S10 Tables S1 and S2 [file bmr.0225.f1.docx]

**Supplementary information for**

**Hsa_piR_016975 is a novel nanotherapeutic target that facilitates hepatocellular carcinoma progression and sorafenib resistance through attenuating Maspin/GPX4-mediated ferroptosis**

**
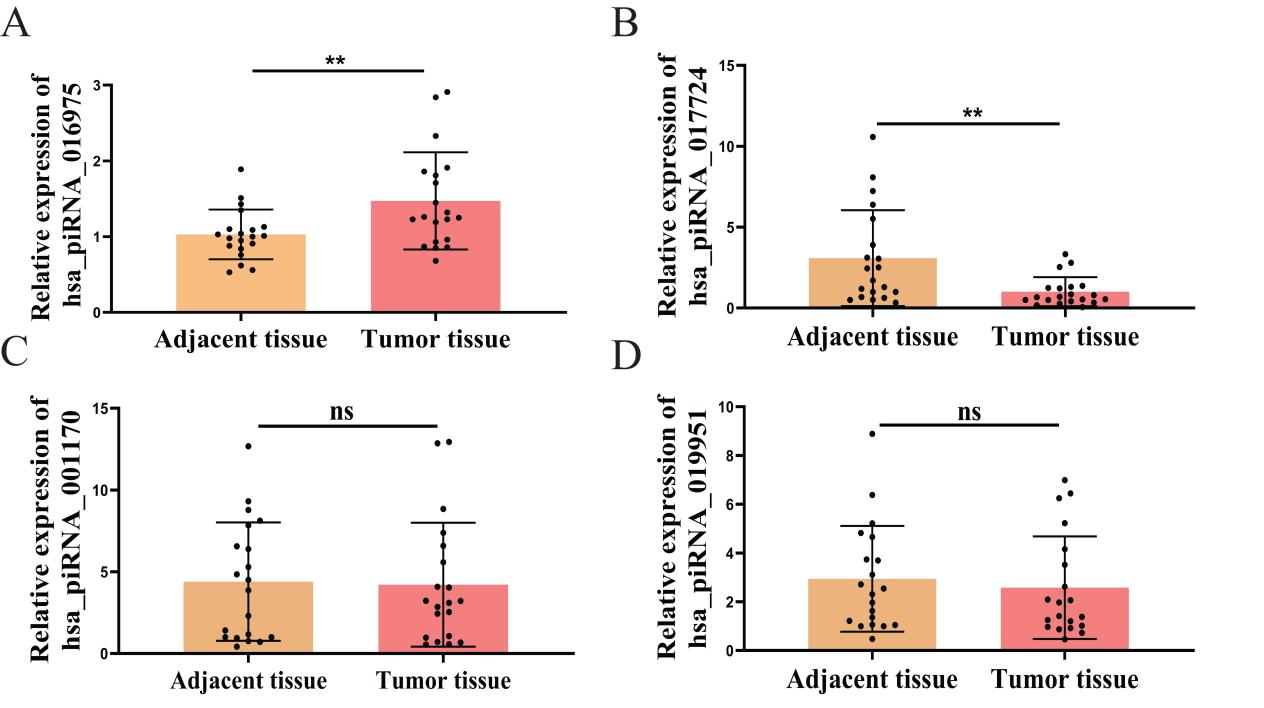
Fig. S1** Detection the expression of four selected piRNAs, hsa_piR_016975 **(A)**, hsa_piR_017724 **(B)**, hsa_piR_001170 **(C)** and hsa_piR_019951 **(D)**, in HCC tissues and their paired adjacent normal tissues by qRT-PCR (n=20). **P*<0.05, ***P*<0.01, ns, not significant.

**
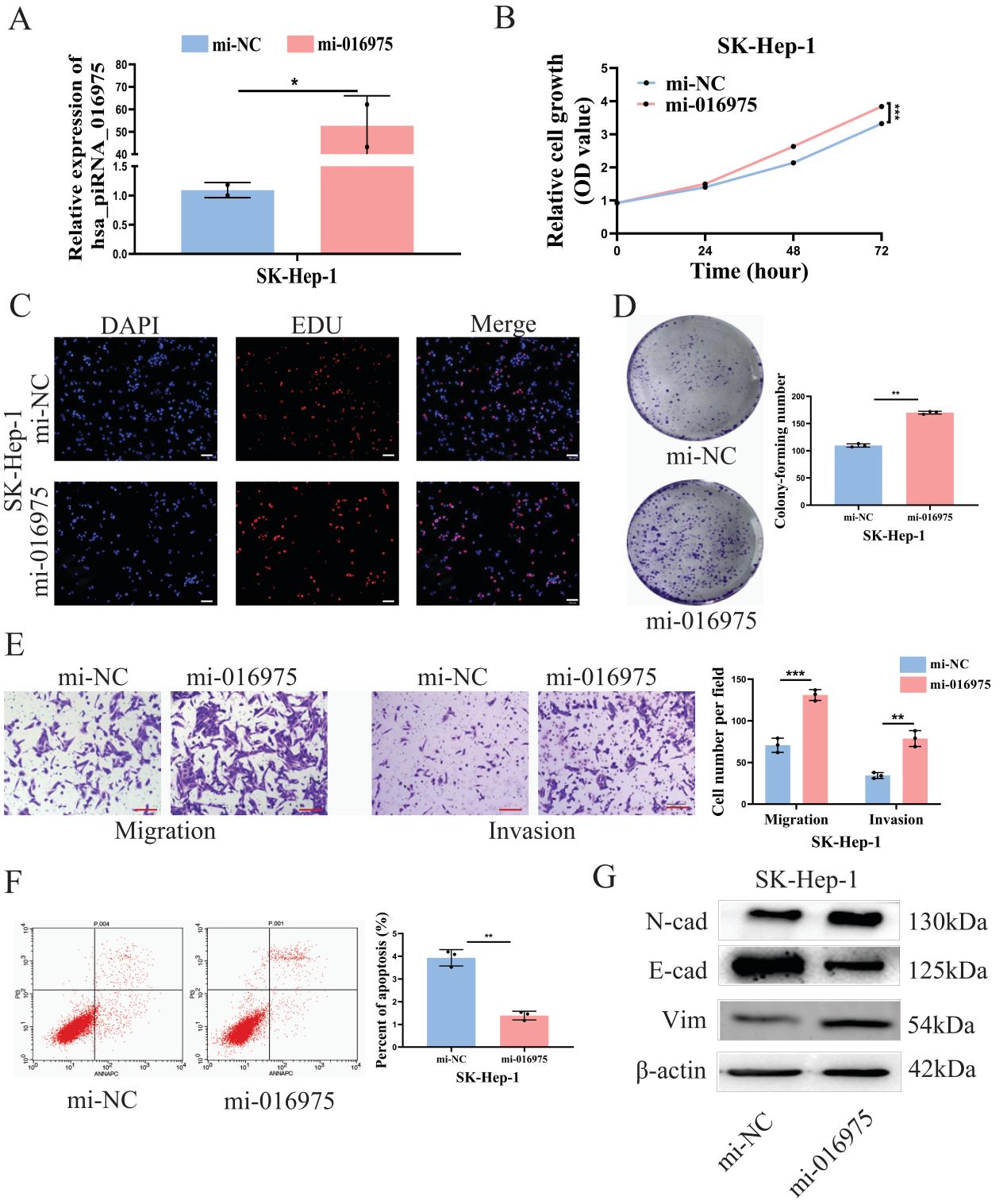
**

**Fig. S2 (A)** Detection of the transfection efficiency of SK-Hep-1 cells treated with hsa_piR_016975 mimics (mi-016975) or empty vector controls (mi-NC). **(B, C, D)** CCK-8, EdU and colony formation assays were performed to analyze the abilities of proliferation in mi-016975 or mi-NC transfected HCC cells (scale bar = 100μm). **(E)** The migration and invasion abilities were detected by transwell assays in mi-016975 or mi-NC transfected HCC cells. **(F)** Flow cytometry was used to analyze the cell apoptosis in mi-016975 or mi-NC transfected HCC cells. **(G)** Westernblot analyzed the expressions of N-cadherin, E-cadherin and Vimentin in mi-016975 or mi-NC transfected HCC cells. **P*<0.05, ***P*<0.01, ****P* < 0.001.

**
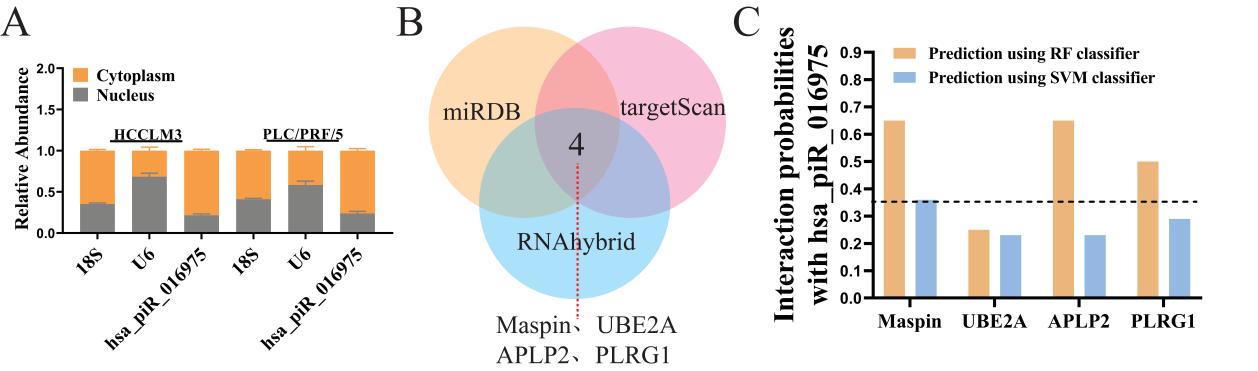
**

**Fig. S3 (A)** The localization of hsa_piR_016975 in HCC cells was detected via a nuclear plasma isolation assay. **(B)** The 4 target genes (Maspin, UBE2A, APLP2 and PLRG1) of hsa_piR_016975 were predicted by miRDB, targetScan databases and RNAhybrid tool. **(C)** RNA-Protein Interaction Prediction (RPISeq) database was used to assess the interaction probability of hsa_piR_016975 with Maspin, UBE2A, APLP2 and PLRG1.

**
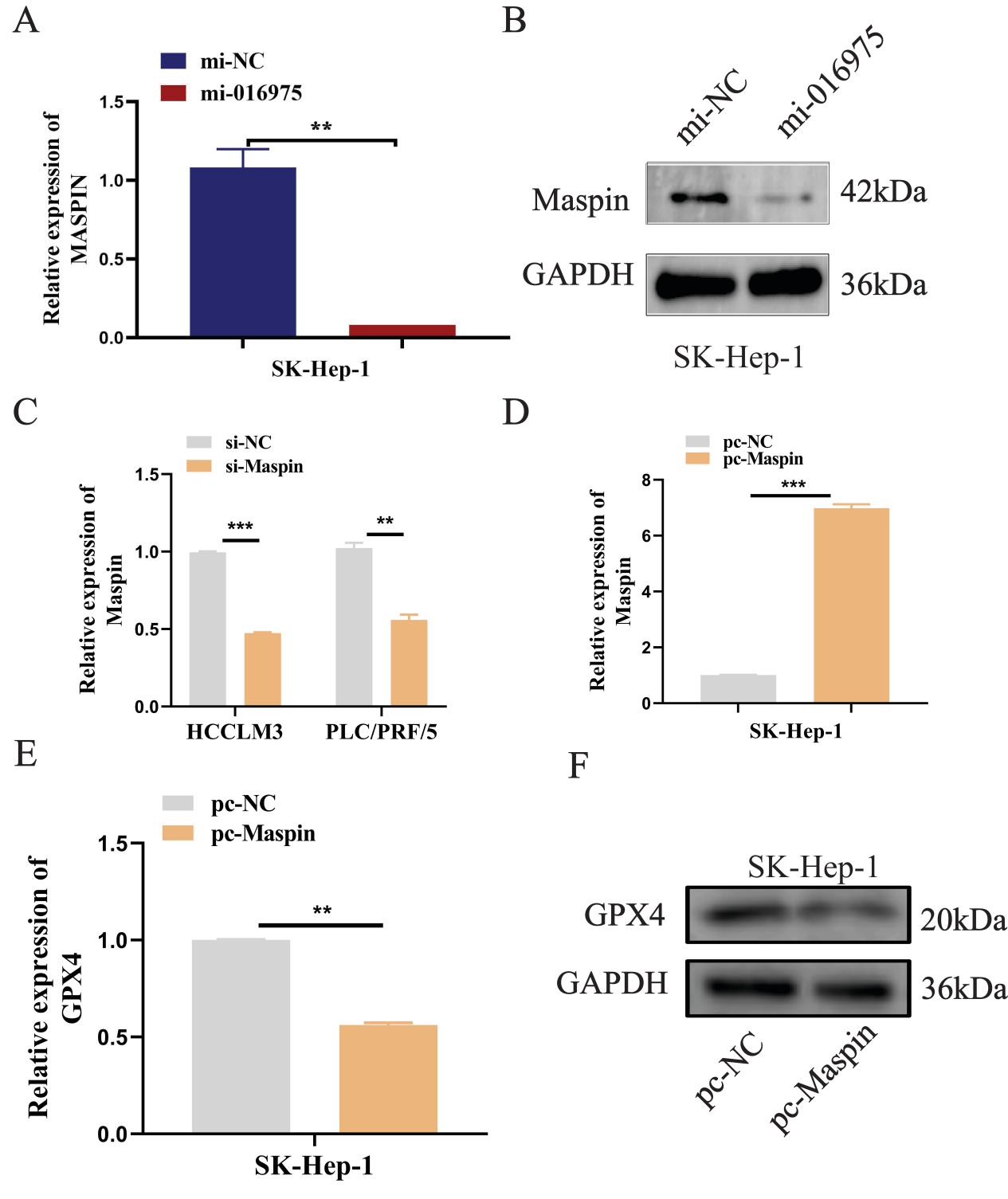
**

**Fig. S4 (A, B)** The mRNA and protein expression levels of Maspin were analyzed in mi-NC or mi-016975 transfected SK-HEP-1 cells. **(C)** Detection the transfection efficiency of HCCLM3 and PLC/PRF/5 cells treated with si-Maspin or si-NC. **(D)** Detection the transfection efficiency of SK-HEP-1 cells treated with pc-Maspin or pc-NC. **(E, F)** The mRNA and protein expression levels of GPX4 were analyzed in pc-Maspin transfected SK-Hep-1 cells. ***P*<0.01, ****P*<0.001.


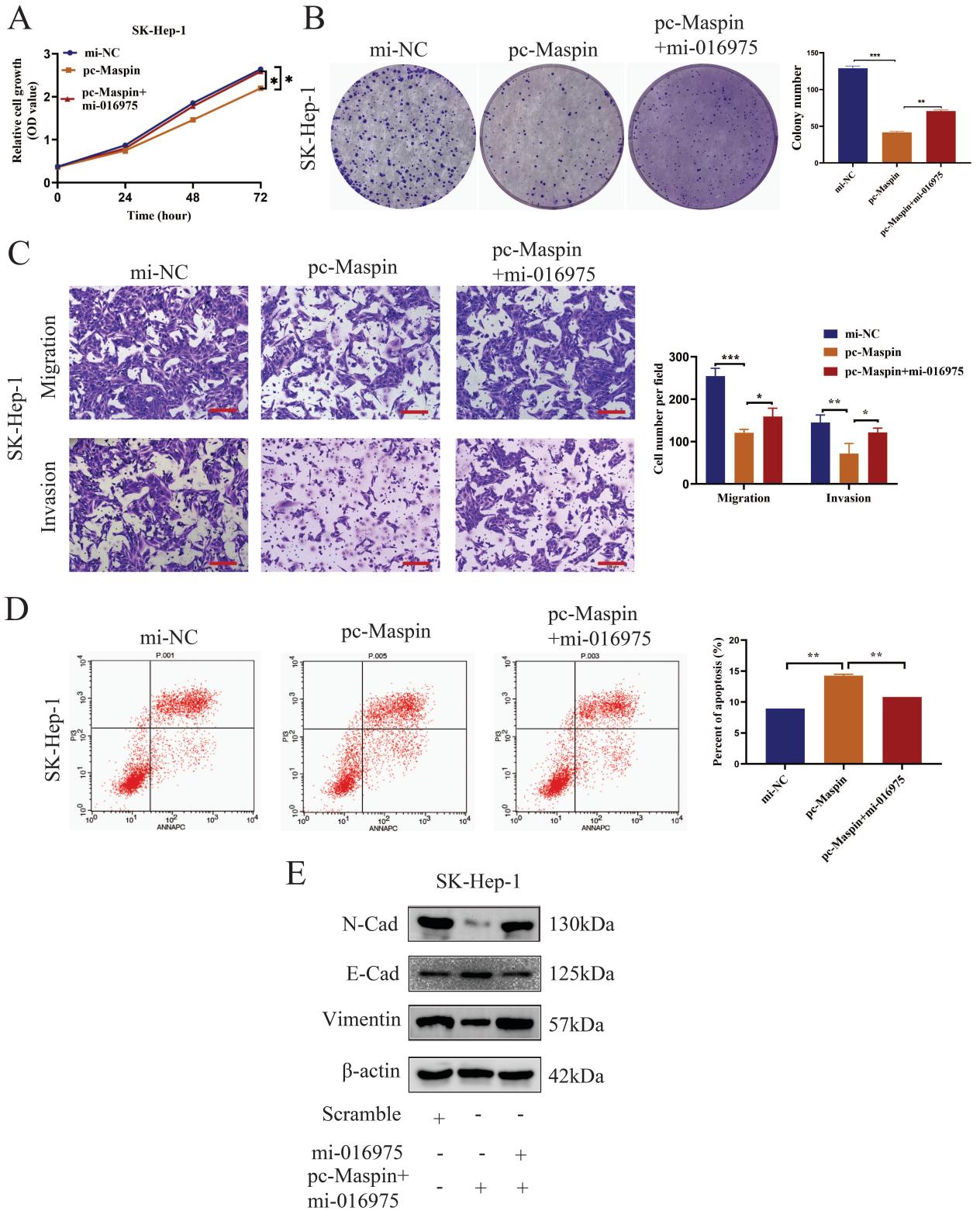


**Fig. S5 (A, B)** CCK-8 and colony formation assays were performed in mi-NC, pc-Maspin or pc-Maspin+mi-016975 co-treated SK-HEP-1 cells. **(C)** The migration and invasion abilities were detectedin in mi-NC, pc-Maspin or pc-Maspin+mi-016975 co-treated SK-HEP-1 cells (scale bar = 100μm). **(D)** Flow cytometry was used to analyze the cell apoptosis in mi-NC, pc-Maspin or pc-Maspin+mi-016975 co-treated SK-HEP-1 cells. **(E)** Westernblot analysis the expressions of N-cadherin, E-cadherin, and Vimentin in in mi-NC, pc-Maspin or pc-Maspin+mi-016975 co-treated SK-HEP-1 cells. **P*<0.05, ***P*<0.01, ****P*<0.001.


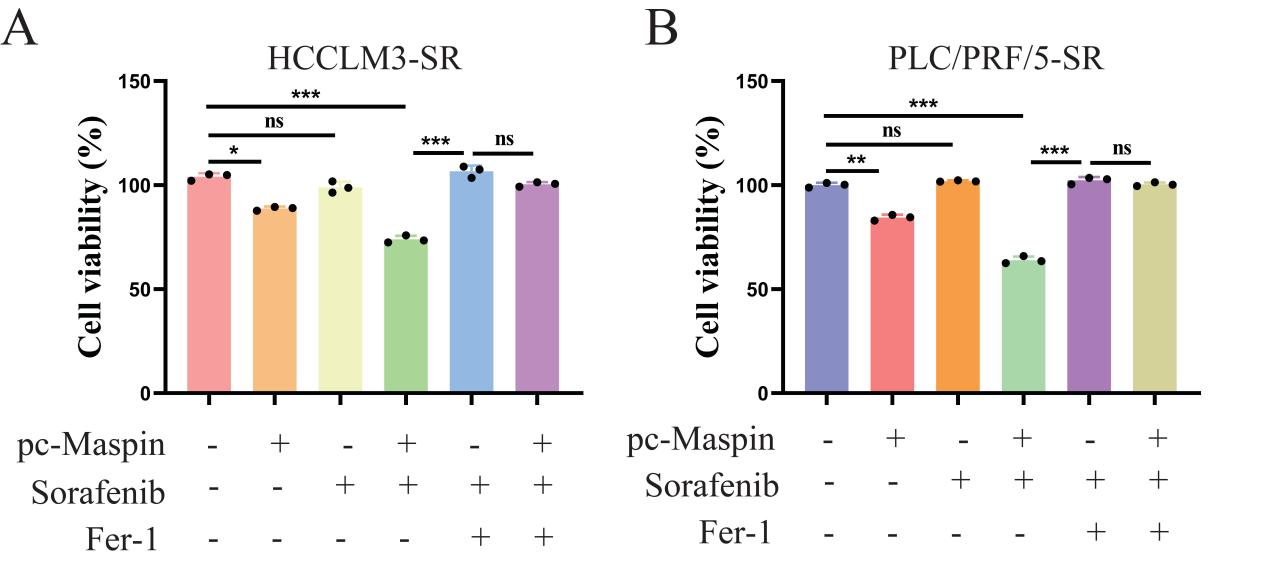


**Fig. S6 (A, B)** Cell viability was analyzed with a CCK-8 kit in pc-Maspin, Sorafenib or Fer-1 co-treated HCCLM3-SR and PLC/PRF/5-SR cells. **P*<0.05, ***P*<0.01, ****P*<0.001, ns, not significant.


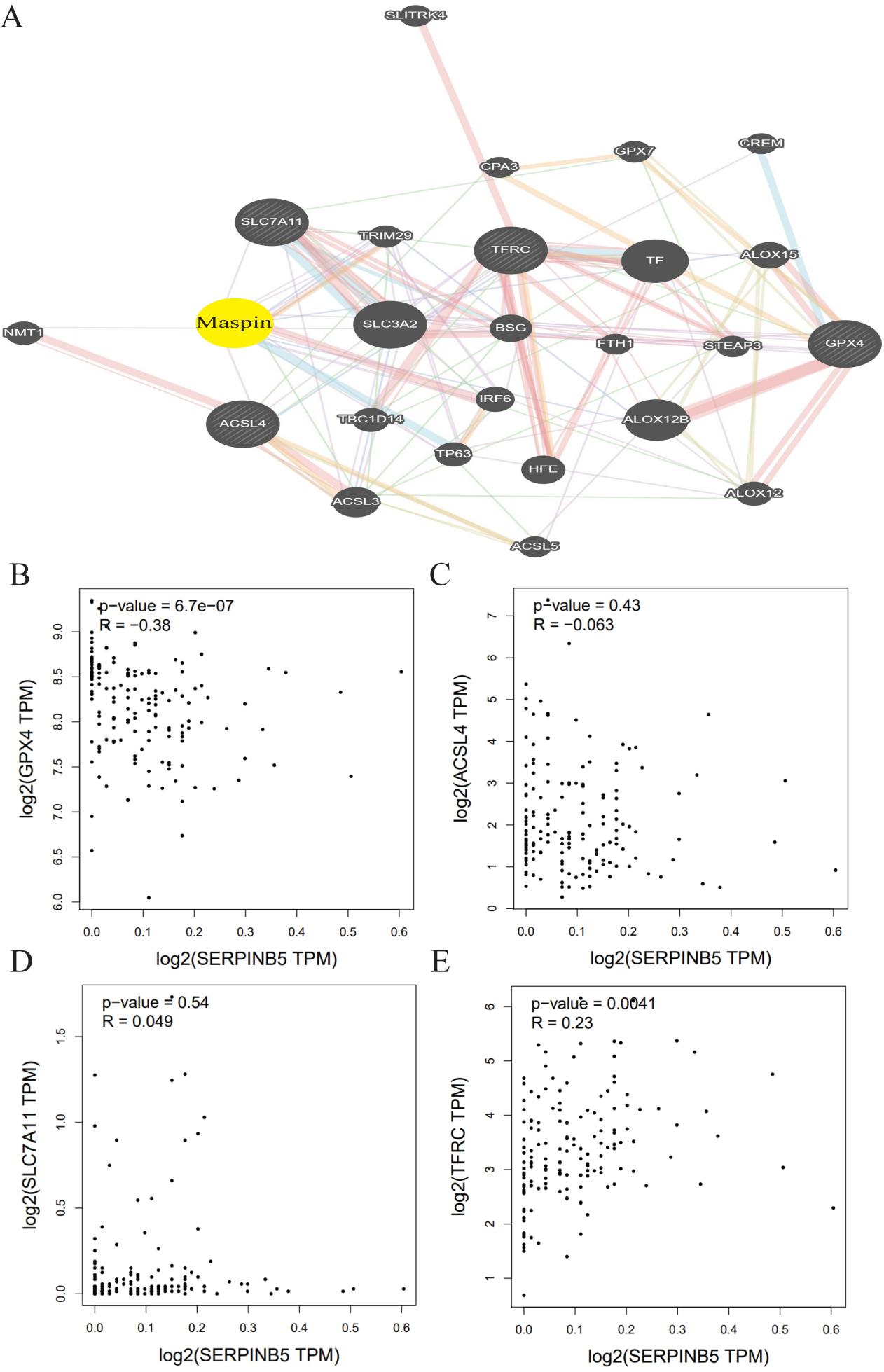


**Fig. S7 (A)** The STRING database predicted the 4 potential target genes (GPX4, ACSL4, TFRC and SLC7A11) of Maspin. **(B, C, D, E)** The Gene Expression Profiling Interactive Analysis database was used to conduct the correlations between Mapin and its targets, GPX4, ACSL4, TFRC and SLC7A11.


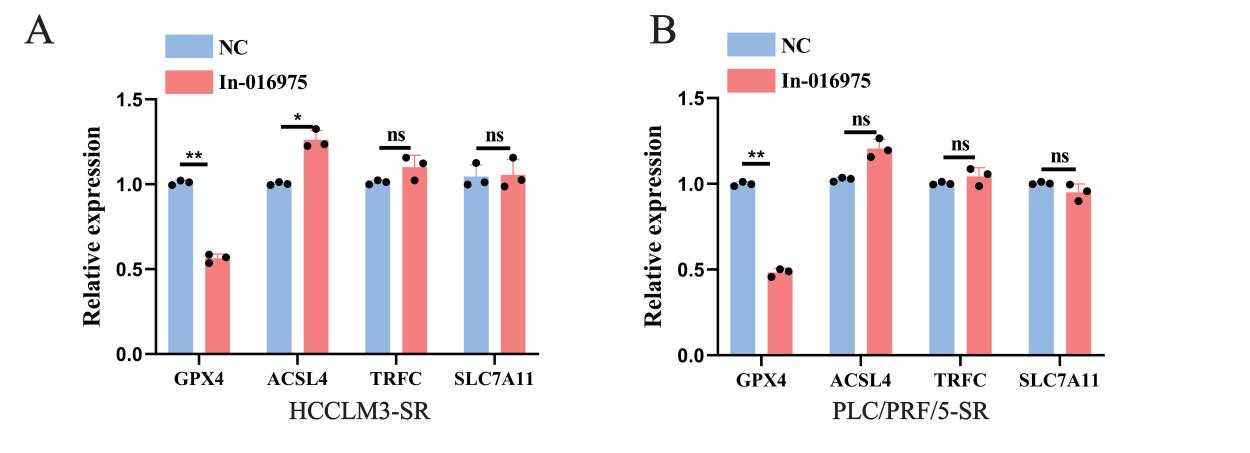


**Fig. S8 (A, B)** Detection of the GPX4, ACSL4, TRFC and SLC7A11 expression in in-016975 treated HCCLM3-SR and PLC/PRF/5-SR cells by qRT‒PCR. **P*<0.05, ***P*<0.01, ns, not significant.


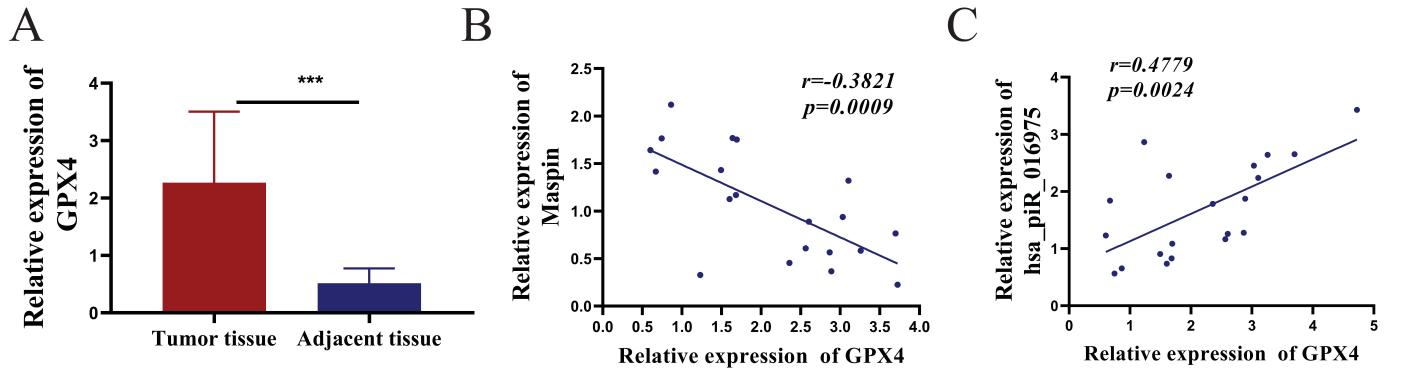


**Fig. S9 (A)** Detection of the GPX4 expression in HCC tissues and paired adjacent normal tissues by qRT‒PCR. **(B, C)** Pearson correlation analysis the relationship between GPX4 and Maspin or hsa_piR_016975 expression in HCC. ****P*<0.001.


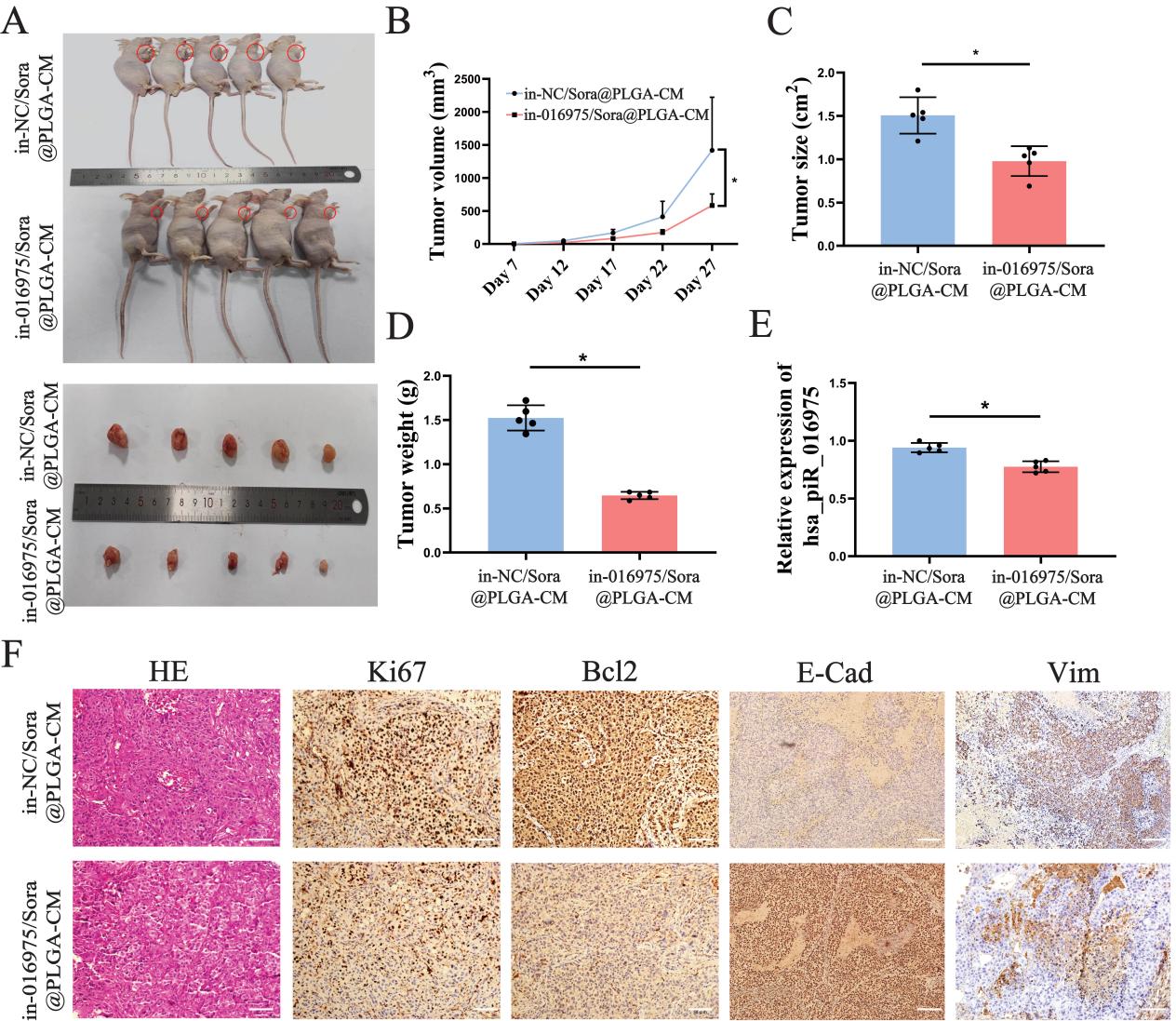


**Fig. S****10** (A) Images of nude mice and transplanted tumors after 27 days of inoculation with in-NC/Sora@PLGA-CM or in-016975/Sora@PLGA-CM treated HCCLM3-SR cells. (B, C, D) The tumor volume growth curve, tumor size and tumor weight were measured in 2 nude mice groups, respectively. (E) Detection of hsa_piR_016975 expression in tumor tissues by qRT‒PCR. (F) Representative images of hematoxylin-eosin (HE) staining and immunohistochemical (IHC) staining for Ki67, Bcl-2, E-cadherin, and vimentin of tumor tissues (scale bar = 50 μm). **P*<0.05.

**Table S1: Association of hsa_piR_016975 expression with clinicopathologic characteristics in patients with HCC**

| **Variables** | **Cases**  **(n = 56)** | **hsa_piR_016975** | | |
| --- | --- | --- | --- | --- |
|  |  | **High**  **(n = 29)** | **Low**  **(n = 27)** | ***P* value** |
| Age (year) |  |  |  | 0.789 |
| ≤59 | 28 | 14 | 14 |  |
| >59 | 28 | 15 | 13 |  |
| Gender |  |  |  | 0.165 |
| Male | 42 | 24 | 18 |  |
| Female | 14 | 5 | 9 |  |
| HBV infection |  |  |  | 0.266 |
| Positive | 49 | 24 | 25 |  |
| Negative | 7 | 5 | 2 |  |
| Hepatocirrhosis |  |  |  |  |
| Present | 51 | 26 | 25 | 0.700 |
| Absent | 5 | 3 | 2 |  |
| Serum AFP (ng/ml) |  |  |  | 0.074 |
| ≤200 | 37 | 16 | 21 |  |
| >200 | 19 | 13 | 6 |  |
| Tumor size (cm) |  |  |  | 0.280 |
| ≤5 | 42 | 20 | 22 |  |
| >5 | 14 | 9 | 5 |  |
| Tumor differentiation |  |  |  | **0.026** |
| Well+Moderate | 10 | 2 | 8 |  |
| Poor | 46 | 27 | 19 |  |
| TNM stage |  |  |  | **0.008** |
| Ⅰ-Ⅱ | 25 | 8 | 17 |  |
| Ⅲ-Ⅳ | 31 | 21 | 10 |  |

**Table S2: The sequences of qRT-PCR primers**

| Name | Sequences (5'-3') |
| --- | --- |
| hsa_piR_016975 | TGGAAGTGGATTTCCGGTGAAGGATGG |
| Maspin Forward Prime | GGAGGCCACGTTCTGTAT |
| Maspin Reverse Prime | CCTGGCACCTCTATGGA |
| GPX4 Forward Prime | GTAACCAGTTCGGGAAGCAG |
| GPX4 Reverse Prime | CACGCAGATCTTGCTGAACA |
| GAPDH Forward Prime | TGCACCACCAACTGCTTAGC |
| GAPDH Reverse Prime | GGCATGGACTGTGGTCATGAG |
| U6 Forward Prime | CAGCACATATACTAAAATTGGAACG |
| U6 Reverse Prime | ACGAATTTGCGTGTCATCC |
